# Microstructured optical fiber based Fabry–Pérot interferometer as a humidity sensor utilizing chitosan polymeric matrix for breath monitoring

**DOI:** 10.1038/s41598-020-62887-y

**Published:** 2020-04-07

**Authors:** Anand M. Shrivastav, Dinusha S. Gunawardena, Zhengyong Liu, Hwa-Yaw Tam

**Affiliations:** 1 Photonics Research Centre, Department of Electrical Engineering, The Hong Kong Polytechnic University, Kowloon, Hong Kong, SAR China; 2 Key Laboratory of Optoelectronic Materials and Technologies, School of Electronics and Information Technology, Sun Yat-sen University, Guangzhou, 510275 China; 3 Photonics Research Centre, The Hong Kong Polytechnic University Shenzhen Research Institute, Shenzhen, China

**Keywords:** Physics, Optics and photonics

## Abstract

This study reports a method for humidity sensing based on a specialty microstructured optical fiber (MOF). A suspended tri-core MOF was fabricated using the stack and draw technique. A low finesse sensing head was prepared by depositing a chitosan polymer matrix within the holes of the MOF, forming a Fabry-Pérot interferometer as a sensing platform while the chitosan film acts as the sensing material. The use of the probe for real-time breath monitoring was also successfully demonstrated. The probe possessed a maximum sensitivity of 81.05 pm/(%RH) for 90–95%RH range while the linear region of the sensor ranged from 70–95%RH. The temperature cross correlation was also experimented, and a lower influence of external temperature was observed. The probe shows an ultrafast response during human breath monitoring with a rising time and recovery time of 80 ms and 70 ms, respectively.

## Introduction

Humidity is an essential criteria that should be accounted for in industrial, agricultural applications as well as in human activities, for example, manufacturing and storage of electronic and computer components, food storage, air conditioning and in pharmaceutical industry^1–6^. Apart from having negative impacts on people with dry skin, low humidity also affects individuals who are suffering from eczema^7^ leading towards increased skin irritations. Furthermore, high humidity may cause discomfort during breathing especially if the person has any chronic lung condition including COPD (Chronic obstructive pulmonary disease) or asthma^8^. In industrial applications, a specific humidity level is required for fabrication of various devices, or it may result in high energy level consumptions^5^. Additionally, humidity sensors are also being used by the European Organization for Nuclear Research (CERN) to monitor experiments in relation to high-energy physics (HEP) at specific humidity levels^9^. Hence, continuous monitoring of air humidity is essential in enhancing human comfort levels as well as in industrial applications.

Over the last few decades, optical fiber technology has significantly evolved and a wide range of physical, chemical and biological sensors have been developed. Many different physical parameters such as displacement, temperature, pressure, refractive index, current, electric field, magnetic field and humidity have been detected using optical fibers either intrinsically or extrinsically owing to their numerous advantages of compactness, immunity to external electromagnetic interference, corrosion resistance, applicability in multiplexing and remote sensing. The detection of relative humidity (RH) using optical fiber as a sensing platform, including evanescent wave sensors^10,11^, fiber Bragg gratings^12,13^, modal interferometers^14,15^ and Fabry-Perot (FP) interferometers^16,17^ have been reported. Among these, FP interferometers are a commonly used transducing platform to realize RH sensing as these are robust, and small in size. FP cavities are also used in the fabrication of a broad range of sensors to monitor pressure^18^, humidity^16^, force^19^, and strain^20^. Suspended core microstructured optical fibers (MOFs) consist of large air holes surrounding a small core which is only a few microns. In such fibers, the core of the fiber is suspended by thin silica struts. Several studies have reported employing suspended-core MOFs for the development of temperature sensors^21^, volatile organic compound (VOC) sensors^22^, displacement sensors^23^, curvature/bend sensors^24^, and humidity sensors^25^ due to their intrinsic features. The special geometry of these fibers facilitate the advantage of strong interaction between the evanescent field and the receptors attached within the holes^22^.

Generally, the working principle of an RH sensor is based on the change in volume and refractive index of the hygroscopic material as they undergo changes in the RH values of the surrounding. Hence, the performance of RH sensors is strongly dependent on the properties of these hygroscopic materials such as polyimide^13^, SiO_2_^16^, SnO_2_^25^, polyvinyl alcohol^14^, agarose^10^, gelatin^11^ and chitosan^17,26^. Among them chitosan based hydrogels have shown great potential in humidity sensing due to its high degree of swelling^27^, reversibility, easy preparation, non-toxicity, and broad range of transparency in light spectrum (300–2700 nm)^28^.

Based on the fiber geometry and types of hygroscopic materials used, a number of studies have reported optical fiber based humidity sensors and a few review articles^6,29^ have also been published summarizing the extensive use of these sensors. The sensors based on evanescent wave spectroscopy are generally designed through tapering the middle section of fiber and deposition of the hygroscopic medium over the sensing medium layer^30^. In another paper reported by Mathew *et al*., buffer-stripped bent SMF together with Polyethylene Oxide (PEO) have been used where PEO acts as the humidity absorbing material^31^. Similarly, there are several other reports on fiber Bragg grating based humidity sensors^32,33^. The sensing mechanism of broadly used FBG based humidity sensors basically relies on the hygroscopic property of the fiber core as it changes its own refractive index. Thus, to realize these sensors, POFs (polymer optical fibers) fabricated using PMMA have been used in various studies^33^. Additionally, a few studies have also reported the use of conventional SMF based on either tilted FBGs or long period gratings^34–36^. The sensors based on these studies are also highly sensitive, but they suffer from a high response time. When considering interferometric sensors, a broad range of sensors have been reported in literatures based on Mach-Zehnder interferometry, Sagnac interferometry and microfiber resonator^37–39^. In a study based on Mach-Zehnder Interferometry, a hetro-core fiber has been spliced between two SMFs where a [poly-glutamic acid/poly-lysine] nanostructured layer-by-layer was deposited over the hetro-core fiber to achieve RH sensing^39^. The sensor operates over the humidity range of 50–92.5% with a power loss of 0.26 dB. The sensor has also shown the possibility of breath monitoring with a response time of ~400 ms.

The present study proposes a versatile RH sensor based on a suspended-core MOF utilizing a chitosan polymer as the receptor. A suspended three-core fiber is used as a platform for preparation of the FP cavity. The air holes of the fiber were filled with the chitosan polymer to execute RH sensing. The sensor probe was tested in a climate chamber with RH value varying from 30% to 95%. Wavelength interrogation method was used by tracking the change in dip wavelength in the interference pattern, which occurs due to the change in volume and refractive index of the chitosan polymer at different RH values. The performance of the sensor was characterized in terms of hysteresis, stability, and sensitivity. Finally, real-time breath monitoring of humans is proposed and validated to demonstrate the industrial applicability of the sensing probe.

## Results and Discussion

### Sensing mechanism and theoretical modelling

**Figure 1:**
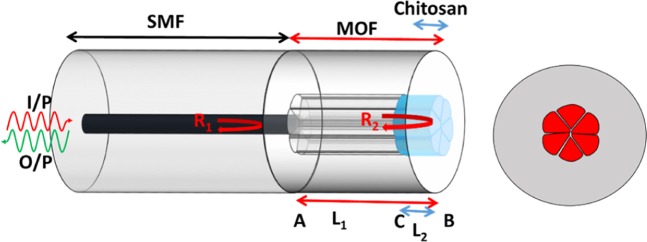
Schematic illustration of the humidity sensor based on a suspended tri-core MOF using a Fabry-Pérot interferometric configuration, and the cross section of the fiber.

Figure 1 represents the schematic illustration of the fabricated probe consisting of a chitosan polymer filled suspended tri-core fiber as the sensing tip. RH sensing of the probe is based on the interaction of humidity (water vapor) with the hygroscopic chitosan polymer via water adsorption causing a change in refractive index as well as an increased volume of the chitosan polymer. These changes result in a shift in the wavelength as well as the power of the reflected light due to the interaction of the evanescent waves at the core-chitosan interface in the FP cavity. Thus, by monitoring the variation in the wavelength of a specific dip, the ambient humidity surrounding the probe can be determined.

**Figure 2:**
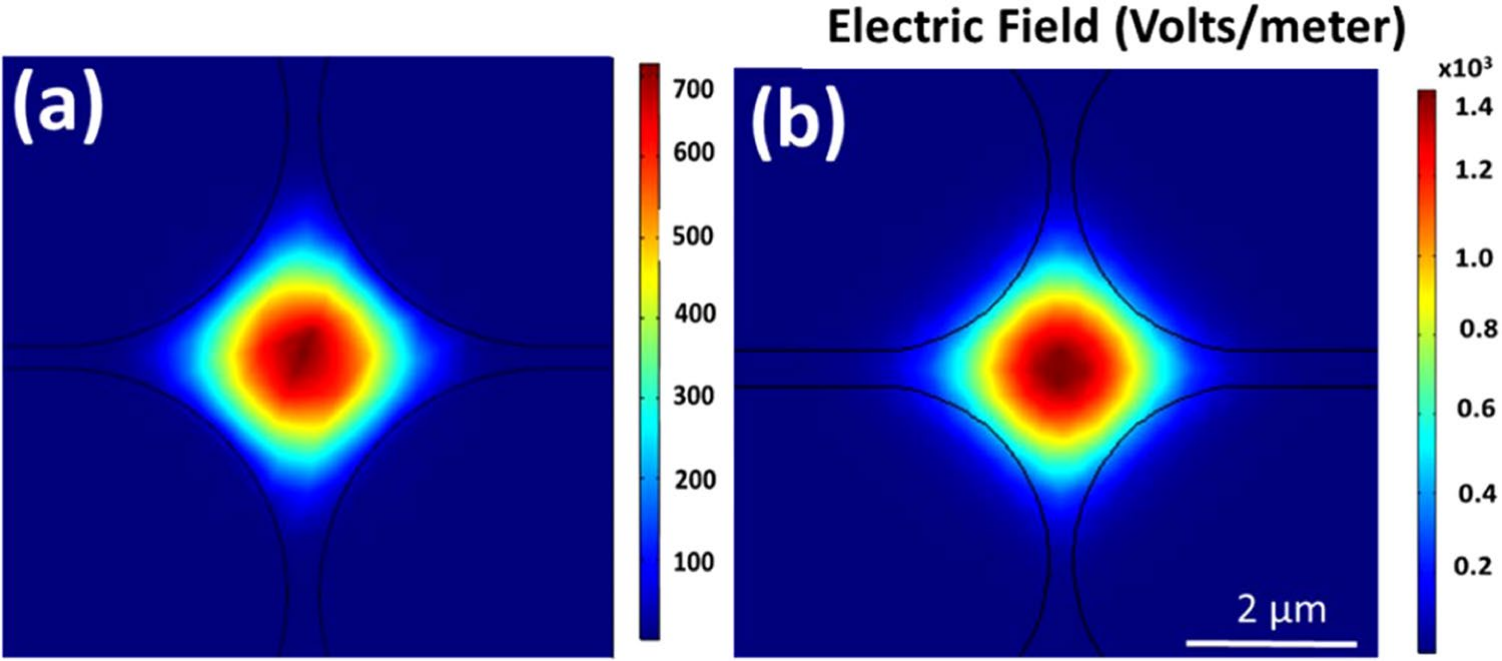
The central element of the fundamental mode of the MOF with (**a**) unfilled and (**b**) filled air holes with chitosan.

Figure 2(a,b) represent the central element of the fundamental mode (LP_01_) of the MOF with the absence of any chitosan filling and with chitosan filling in the air holes, respectively, which were simulated by the finite element method (FEM) using COMSOL at 1440.6 nm wavelength. The figures depict that in the presence of chitosan in the air holes, the light guided through the fiber core interacts with the chitosan region by the evanescent field which plays an important role in humidity sensing. The refractive index of the chitosan was considered as 1.43^17^.

The MOF with and without the chitosan polymer filling belongs to a single fiber optic cavity but with different effective indices, since the fundamental mode of light will propagate only through the central core with the absence of any reflective surface in the fiber core. Hence, in theoretical analysis of the effect of the chitosan polymer, the effective refractive index of the bare MOF region is considered as *n*_mof_ while the effective refractive index of the chitosan filled regime is *ɳ*_c_. If the fiber optic cavity possesses a length of *L*_1_, while the length of the chitosan filled region is *L*_2_, then the effective index of the cavity can be calculated as:

1 $${n}_{eff}=\left[\frac{({L}_{1}-{L}_{2}){n}_{\text{mo}f}+{L}_{2}{n}_{c}}{{L}_{1}}\right]$$

Moreover, if the effective refractive index of the SMF region and outer medium are *n*_smf_ and *n*_out_, respectively then, according to Fresnel’s equation, the reflected intensities at SMF-MOF region and MOF-outer region which are *R*_1_ and *R*_2_, respectively can be denoted as:

2 $${R}_{1}={\left[\frac{{n}_{smf}-{n}_{eff}}{{n}_{smf}+{n}_{eff}}\right]}^{2},\,{R}_{2}={\left[\frac{{n}_{eff}-{n}_{out}}{{n}_{eff}+{n}_{out}}\right]}^{2}$$

and the phase delay in light due to the fiber optic cavity can be expressed as:

3 $$\phi =\frac{4\pi {n}_{eff}{L}_{1}}{\lambda }$$

If *α* represents the intensity attenuation factors and *γ*,the optical transmission loss factor for the FP cavity then, by taking into account the Eqs. (2) and (3), the normalized reflected intensity can be expressed as^17^:

4 $${R}_{FP}={R}_{1}+{(1-\alpha )}^{2}{(1-\gamma )}^{2}{(1-{R}_{1})}^{2}{R}_{2}-2\sqrt{{R}_{1}{R}_{2}}(1-\alpha )(1-\gamma )(1-{R}_{1})\cos \,\phi $$

Hence, by calculating the effective refractive index of the chitosan filled regime and its change with humidity, the shift in the interference pattern can be observed using Eq. (4).

Additionally, it should be noted that the reduced dimensions of the three fiber cores and the high numerical aperture that exists owing to the difference in the effective refractive index between the fiber core and the air cladding results in an increased evanescent field^40,41^. Furthermore, the three cores provide sufficient surface area for the deposition of the chitosan material as well.

### Humidity sensing response

**Figure 3:**
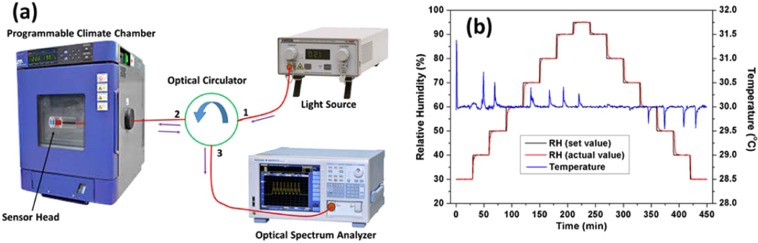
(**a**) Experimental setup for the humidity sensing experiments and (**b**) calibration curves of humidity and temperature in the climate chamber during the experiments.

Figure 3(a) shows the experimental setup used to monitor the sensing performance of the probe towards humidity. The sensing probe was fixed inside a programmable humidity chamber and the humidity was varied from 30% to 95% at a constant temperature of 30 ± 1.5 °C. The set values and the actual values of humidity measured by the inbuilt hygrometer within the chamber, are shown in Fig. 3(b). Subsequently, light was launched into the probe via a broadband source (1420 nm-1600 nm) and an optical circulator while the reflected spectra were recorded by an optical spectrum analyzer (OSA). The resolution of OSA used during the experiment is kept at 0.02 nm.

**Figure 4:**
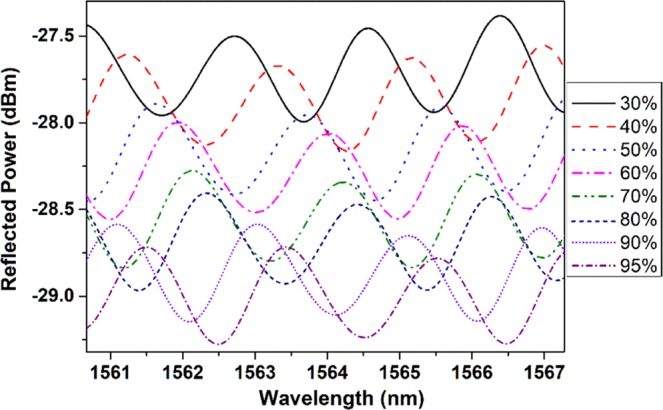
Measured output spectra of the probe at different humidity values in the climate chamber.

Figure 4 shows the measured output spectra of the probe at different humidity values in the climate chamber. A shift in the interference pattern can be observed from Fig. 4, which occurs due to the change in refractive index of the chitosan polymer matrix as the humidity of the climate chamber varies.

## Characterization of the humidity sensor

### Probe calibration

**Figure 5:**
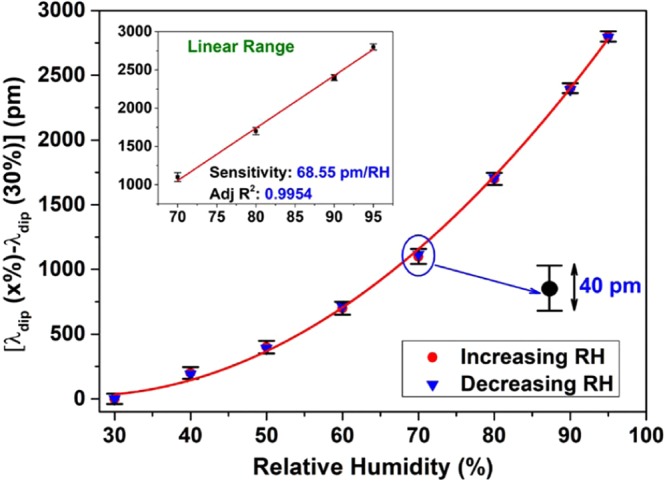
Measured wavelength shift of a specific dip as a function of relative humidity of the environment. The inset shows the linear response of the sensing probe.

The sensor probe was calibrated by tracing a specific dip wavelength (1661.5 nm) of the interference pattern. The results are shown in Fig. 5 which indicates a total shift of 2.8 nm for a humidity level varying from 30% to 95%. Furthermore, the hysteresis behavior of the same probe was investigated by repeating the measurement for five times with increasing and decreasing humidity, inside the climate chamber. The standard deviation of the wavelengths at different humidity values was 40 pm, which is represented as error bars in Fig. 5. The maximum sensitivity of the probe was 81.05 pm/(%RH) over a range of 90–95%RH. From Fig. 5, it can be observed that the probe possesses a non-linear behavior due to the presence of the chitosan polymer and also has a non-linear hygroscopic response^28^. At low RH values, the chitosan polymeric film is nearly at a dehydration state resulting in a less sensitivity whereas at high RH values, the chitosan starts to absorb more moisture causing a larger change in the refractive index as well as the thickness of the chitosan film making the probe more sensitive. The inset in Fig. 5 represents that the probe exhibits a liner response with a humidity change from 70% to 95%, showing a sensitivity of 68.55 pm/(%RH) which verifies the applicability of the probe in high humidity conditions.

### Temperature dependence

**Figure 6:**
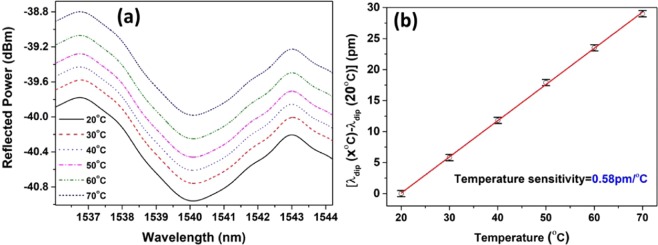
(**a**) Spectral response and the (**b**) temperature sensitivity of the probe at different temperatures at 50%RH.

In addition to the humidity characteristics, the behavior of the probe was also investigated with varying ambient temperature from 20 °C to 70 °C at a constant humidity of 50%. The response of the probe for the aforementioned temperature range is shown in Fig. 6(a) and the temperature calibration measurement is shown in Fig. 6(b). It can be clearly observed that the probe has a temperature sensitivity of only 0.58 pm/°C, which is considerably less when compared to its sensitivity towards humidity. The total shift obtained for the temperature range of 20 °C to 70 °C is ~29 pm which exhibits an error of less than 0.5%RH. Therefore, the temperature effect of the proposed humidity sensor can be ignored in the operation range of 20 °C to 70 °C. The change in reflected power is due to the fluctuations in the input light during the experiments, which is also noticeable in the characteristic curve of the sensing probe (Fig. 4).

### Stability of the sensor

**Figure 7:**
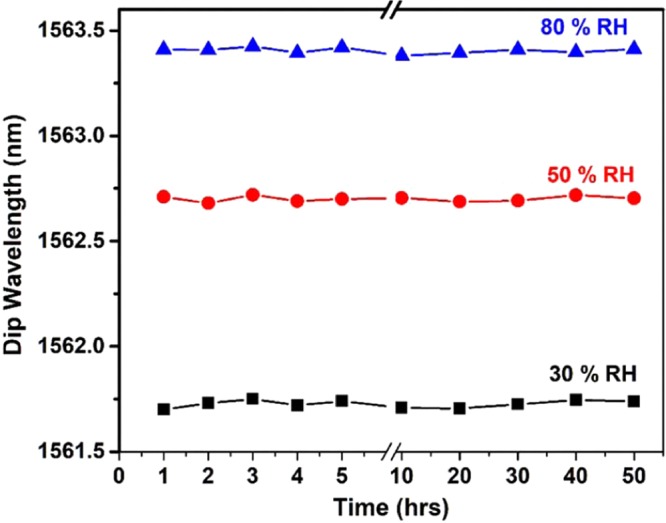
Stability of the humidity sensor measured at 30%, 50% and 80%RH over 2 days.

The long-term stability of the fabricated probe was explored by exposing the probe to different humidity conditions (i.e. 30%, 50% and 80%) for two days. The variation in the dip wavelengths at different humidity values are plotted as a function of time in Fig. 7, which confirms a strong long-term stability of the sensing probe. The fluctuations in the dip wavelengths for each humidity value is within a measurement error value of 40 pm (about 0.5%RH) which is similar to that observed during the probe calibration process.

## Breath Measurements

**Figure 8:**
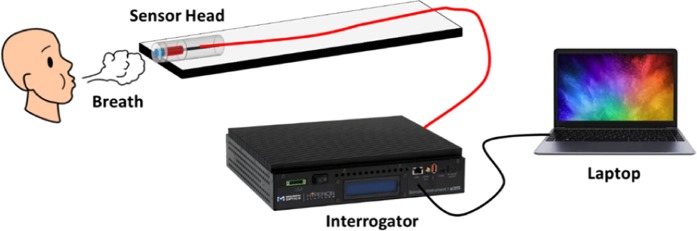
Experimental setup for human breath monitoring.

To demonstrate the capabilities and potential application of the sensor, it was tested with real-time monitoring of human breath. Figure 8 illustrates the experimental setup to monitor the breath. The probe was fixed on a solid substrate with an adhesive polyimide tape and the output end of the probe was connected to an optical interrogator (Micro Optics: si255) which can detect the change of the dip wavelength as a function of time.

The sampling rate of the interrogator is 5 kHz and the data were collected through a laptop to record the output dip wavelength. During the test, the sensing head was placed in close proximity to the mouth of a person and a variation in the surrounding humidity was observed due to the continuous exhalation.

**Figure 9:**
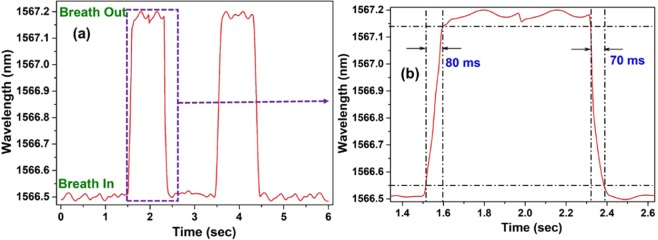
(**a**) Response of the sensor to human breath containing two cycles of exhalation and inhalation and (**b**) detailed analysis of human breath for one cycle.

Figure 9(a) shows the wavelength change of one dip during two cycles of breath, including exhalation and inhalation. From the result, it can be clearly seen that the dip wavelength increases during exhalation, and it decreases to the baseline during inhalation. Furthermore, from the result of one cycle of exhalation as shown in Fig. 9(b), it can be observed that the response and recovery time of the sensor are ~80 ms and ~70 ms, respectively.

## Comparison with other studies

Table 1 shows a comparison of the performance of the proposed probe with respect to previously reported research studies for humidity sensing. A fiber optic Sagnac interferometer has also been proposed by Chen *et al*. using chitosan as the hydroscopic material^38^ where the sensor possessed a sensitivity of 81 pm/%RH which is relatively close to our proposed sensor. However, the study does not demonstrate any real time experiment. It is evident that the proposed sensor possesses the quickest response time along with a moderate sensitivity, demonstrating its industrial applicability for real time breath monitoring. Liu *et al*., has reported a Fabry-Perot humidity sensor using chitosan as a water-absorbing receptor to achieve humidity sensing. Even though, the sensitivity of this sensor is better than the proposed sensor, it suffers from a high response time (around 60 seconds) and the study does not demonstrate any industrial application. Although, chitosan has been integrated with optical fibers to realize humidity sensing^17,26,38^, as demonstrated in our current research study, a combination of micro-structured fiber with chitosan is useful in providing a fast and moderately sensitive response, in real-time breath monitoring which highlights its advantages as a potential candidate for breath monitoring.

**T.able 1:** Performance comparison of the proposed sensor and other optical fiber-based humidity sensors reported in literature.

Sensing Material	Optical fiber	Sensing method	Range (%RH)	Sensitivity (Resolution)	Response time	Application
Gelatin^11^	Tapered optical fiber	Interferometric	9–94	0.016 dBm/%RH	70 ms	
TiO_2_/SnO_2_^12^	Long Period Grating	Long Period Grating	40–95	221 pm/%RH	—	
Silica/di-ureas^32^	Fiber Bragg grating	Fiber Bragg grating	5–95	22.2 pm/%RH		Civil Engineered Structure Monitoring
PMMA polymer^33^	Fiber Bragg grating	Etched Polymer Optical Fiber	30–90	33.6 pm/%RH	7 min	
Polyvinyl Alcohal^35^	Long Period Grating	Long Period Grating	33–97	~5680 pm/%RH	<1 min	Structural Health Monitoring
Polyvinyl Alcohol^43^	Tilted Fiber Bragg Grating	Tilted Fiber Bragg Grating	20–98	14.95 dBm/%RH	—	
Calcium Chloride^34^	Air gap Long Period Grating	Air gap long period grating	50–95	1350 pm/%RH		
SiO_2_ –Nanospheres^36^	Long Period Grating	Long Period Grating	20–50 50–80	63.33 pm/%RH 451.78 pm/%RH	30 ms (rise) 153 ms (recovery)	
[PDDA/Poly R-478] nanostructured^30^	Tapered Fiber	Evanescent wave	75–100	16 dBm/%RH	300 ms	
Polyethylene Oxide (PEO)^31^	Buffer-striped 1060XP Fiber	Evanescent wave	80–95	1 dB/%RH	760 ms	Breath Monitoring
No coating^37^	Silica/polymer Microfiber	Knot Resonator	17–98	8.8 pm/%RH (0.017%)		
[poly-glutamic acid/poly-lysine]^39^	Hetro-core Fiber	Modal Interferometric	50–92.9	0.0052 dB/%RH	400 ms	Breath Monitoring
Polyvinyl Alcohol^14^	Photonic Crystal Fiber	Modal Interferometric	20–95	40.9 pm/%RH	—	
(P_4_VP·HCl/PVS)_10_^15^	Thin Core Fiber	Modal Interferometric	20–90	84.3 pm/%RH (0.78%)	2 sec (rise) 10 sec (fall)	
Chitosan^38^	Polarization Maintaining Fiber	Sagnac Interferometer	20–95	81 pm/%RH (2.04%)		
Ti_3_O_5_/SiO_2_^16^	Single Mode Fiber	Fabry-Perot Interferometer	1.8–74.7	430 pm/%RH	5 sec	
Chitosan^26^	Hollow-Core PCF	Fabry-Perot Interferometer	20–95	130 pm/%RH	380 ms	
(PAH/PSS)_15_^44^	Hollow-Core PCF	Fabry-Perot Interferometer	5–90	0.08 dB/%RH (0.125%)	2 sec (rise) 6 sec (fall)	
Chitosan^17^	Hollow-Core PCF	Fabry-Perot Interferometer	35–95	280 pm/%RH (0.02%)	<60 sec	
SnO_2_^25^	Twin Suspended Core Fiber	Fabry-Perot Interferometer	20–90	0.14 rad/%RH	370 ms (rise) 380 ms (recovery)	Breath Monitoring
Chitosan (*Present Study)*	Suspended tri-core Fiber	Fabry-Perot Interferometer	30–95	81.05 pm/%RH (0.5%)	80 ms (rise) 70 ms (recovery)	Breath Monitoring

## Conclusion

In conclusion, a Fabry-Perot interferometer based on a homemade suspended tri-core fiber was successfully employed for humidity sensing using the hygroscopic property of chitosan polymeric matrix. The sensor relies on the change in the refractive index of the chitosan polymer which in turn causes a phase change in the reflected light at different surfaces that can be observed by a shift in the interference pattern. In the current study, a shift of 28 pm was observed over a climate humidity level ranging from 30–95%. The probe possessed a linear response over the humidity range from 70–95%. The sensor exhibits a maximum sensitivity of 81.05 pm/(%RH) (for the high humidity ranges from 90–95%) and a maximum deviation of 40 pm, showing an error of only ±0.5%RH. Further performance characteristics of the sensor in terms of hysteresis, stability and influence of the temperature were also analyzed. In an effort to demonstrate the applicability of the proposed sensor in the biomedical industry, the probe was used to monitor human breath where it indicated an extremely fast response without any drift in the baseline. Therefore, such a sensor is promising in biomedical applications such as monitoring breath of individuals suffering from asthma and any lung disease in addition to monitoring humidity levels in agriculture and food storage units where maintaining a certain humidity level is of utmost importance.

## Methods

### Characterization of the suspended-core MOF

**Figure 10:**
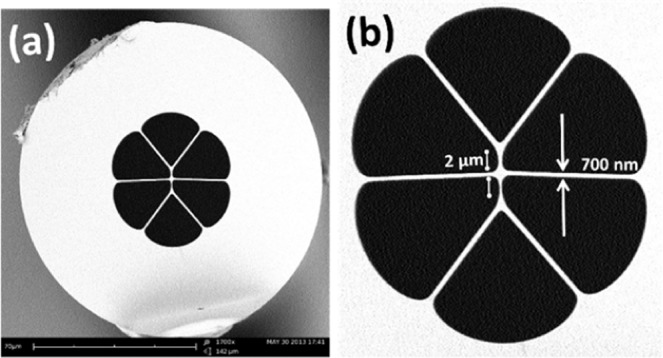
Scanning Electron Microscopic (SEM) images of the cross section of the MOF at (**a**) 1700X and (**b**) 4000X magnification.

The suspended-core MOF consisting of three cores which are suspended by thin silica struts and surrounded by six large air holes was designed and fabricated using the stack and draw technique^42^. Figure 10 shows the cross-sectional SEM images of the MOF, where the cores of the fiber are ~1.5 µm in diameter and separated by about 2 µm from each other. The thickness of the silica struts was ~700 nm. The MOF possesses several unique advantages due to its special structure. For example, the large air holes make it easier to deposit various gas sensitive/chemically sensitive materials on the core region, providing strong interaction between the materials and the light guided in the core. Additionally, the reduced diameter of the fiber core and large air holes lead to a stronger evanescent field as discussed in literature^41^.

### Fabrication of the sensor

**Figure 11:**
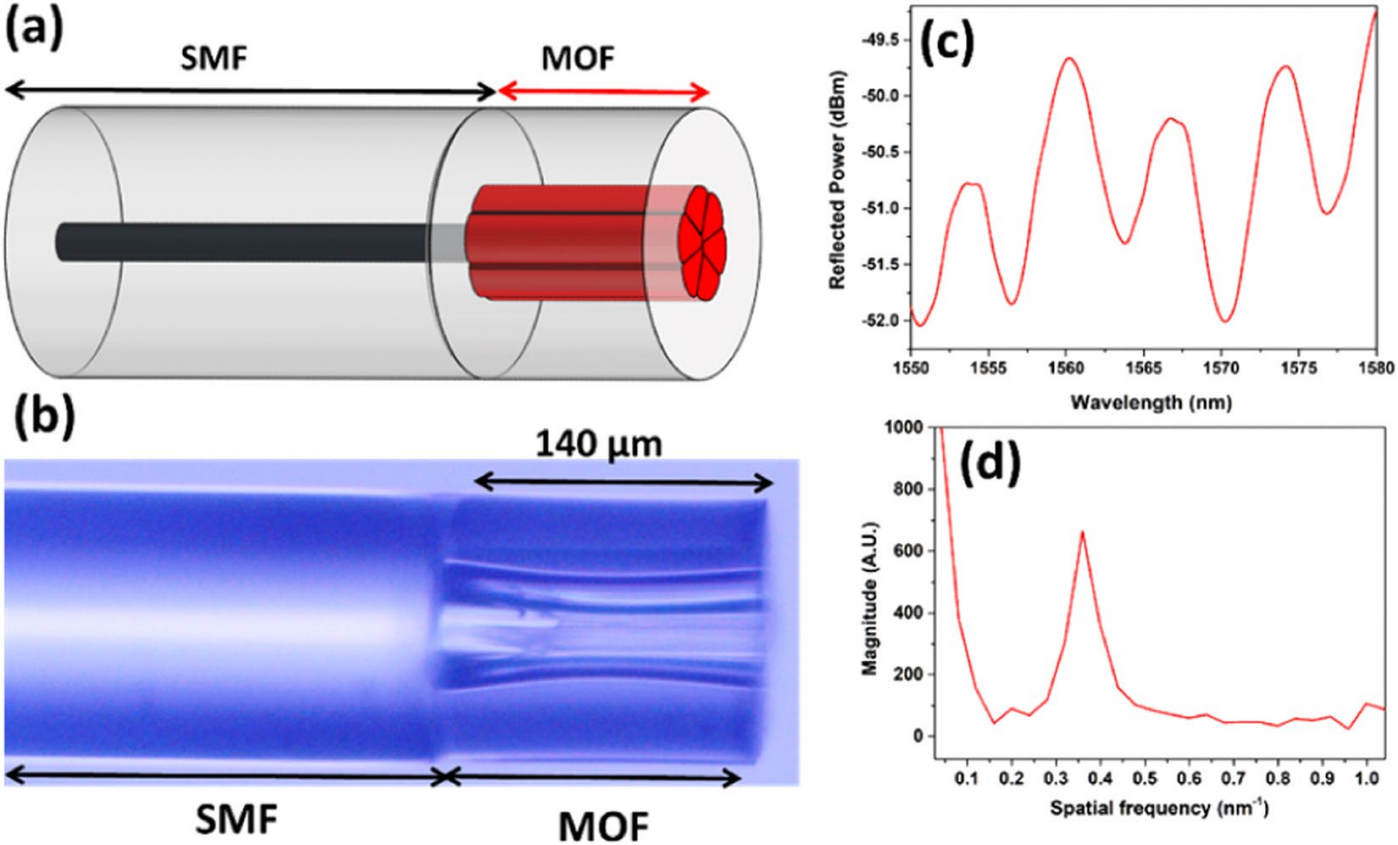
(**a**) Schematic figure and (**b)** microscopic image of the SMF-MOF probe. (**c**) Reflected interference pattern and (**d**) corresponding FFT spectrum of the SMF-MOF probe.

The proposed FPI sensor for humidity sensing was prepared by splicing a conventional single mode fiber (SMF-28) with a suspended three-core MOF. The schematic representation and the microscopic image of this structure are shown in Fig. 11(a,b), respectively. The end of the fiber was perfectly cleaved. Afterwards, a Fitel 175 splicer was used to splice SMF and MOF using a customized splicing program since MOF exhibits large air holes and very thin silica struts. The corresponding interference pattern and the FFT spectrum are shown in Fig. 11(c,d), respectively. The length of the suspended core MOF was maintained at 140 µm.

A humidity sensitive chitosan polymer was synthesized and part of the air holes of the MOF was filled with the polymeric solution to create a thin film at the sensing surface. The polymer was synthesized by mixing 1 wt% chitosan powder, (90–95% deacetylation degree and 50–800 mPa·s viscosity) in acetic acid solution (4% v/v) using a magnetic stirrer for 24 h at room temperature (25 °C). Then, the solution was filtered through a filter paper having a 50 µm mesh size. Finally, the tip of the SMF-MOF fiber was dipped vertically in the solution for 30 seconds and then the probe was dried at room temperature for 24 h. This completed the probe fabrication process. The schematic diagram of the final probe is shown in Fig. 1. Measurement of the exact length of the chitosan filled region is rather challenging. However, the approximate length of the chitosan filled regime (1) can be calculated by the capillary action relation:

5 $${\rm{l}}=\frac{2{\rm{\gamma }}\,\cos \,{\rm{\theta }}}{{\rm{\rho }}{\rm{gr}}}\,$$

where, ${\rm{\gamma }}$ denotes the surface-tension at chitosan-matrix and air interface, ${\rm{\theta }}$ is the contact angle, ${\rm{\rho }}$ and ${\rm{g}}$ represents the chitosan polymer density and gravitational velocity, respectively while r is the radius of the air-hole (around 35 µm).
